# Percutaneous Coronary Intervention for Coronary Arteries with Origin and Course Anomalies: Case Reports and Literature Review

**Published:** 2019-07

**Authors:** Murat Akcay, Ilkay Camlıdag, Okan Gulel

**Affiliations:** 1 *Department of Cardiology, Faculty of Medicine, Ondokuz Mayıs University, Samsun, Turkey. *; 2 *Department of Radiology, Faculty of Medicine, Ondokuz Mayıs University, Samsun, Turkey.*

**Keywords:** *Coronary vessels*, *Coronary artery diseases*, *Percutaneous coronary intervention*

## Abstract

Coronary artery anomalies (CAAs) are defined as variants of normal epicardial coronary arteries. They are mostly detected incidentally during coronary angiography. Clinical studies have shown that abnormal origins and courses of coronary arteries make them more prone to atherosclerosis. Percutaneous treatment for atherosclerotic occlusions in anomalous coronary arteries has some difficulties, including inadequate guiding-catheter support and the need for an experienced operator. Here, we describe successful percutaneous coronary interventions for critical stenoses in 2 different CAAs and present a brief literature review.

## Introduction

Coronary artery anomalies (CAAs) are defined as variants of normal epicardial coronary arteries. They are most often classified as abnormalities of origination, course, termination, and intrinsic structure.^1^^-^^4^ CAAs are mostly detected incidentally during coronary angiography, with a rate of 0.6% to 1.3% in various series.^1^^, ^^2^ Clinical studies have shown that abnormal origins and courses of coronary arteries render them more prone to atherosclerosis.^3^ In addition, an abnormal course between the pulmonary artery and the aorta may lead to myocardial ischemia and sudden death.^1^^, ^^2^^, ^^4^ Here, we describe successful percutaneous coronary interventions for critical stenoses in 2 different CAAs and present a brief literature review.

## Case Reports

**Figure 1:**
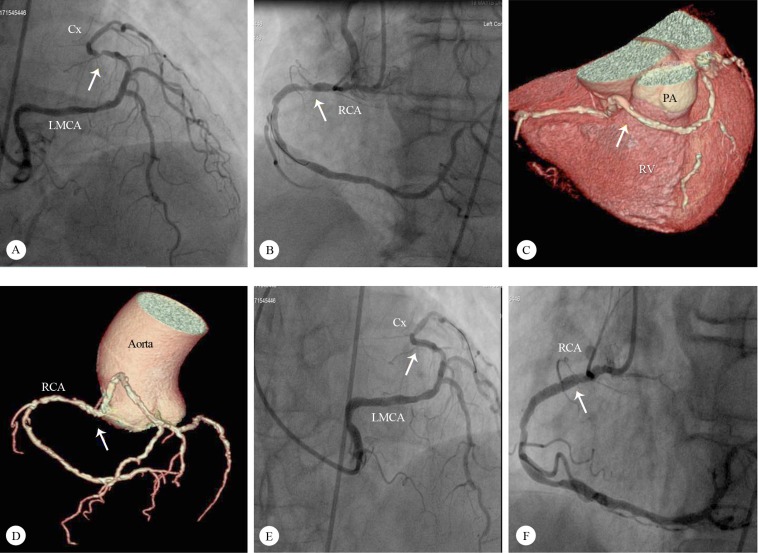
A) Left anterior oblique cranial angiographic view of the LMCA originating from the right sinus of Valsalva and a severe midportion LCx stenosis (arrow indicates the stenosis); B) Left anterior oblique angiographic view of a severe proximal RCA stenosis (arrow indicates the stenosis); C) Computed tomographic anterolateral angiographic imaging of the LMCA originating from the right sinus of Valsalva (arrow indicates the LMCA course); D) Computed tomographic anteroposterior angiographic imaging of the origin and course of the LMCA and the RCA (arrow indicates the LMCA and RCA origin); E) Left anterior oblique cranial angiographic view of the successful percutaneous intervention on the midportion of the LCx (arrow indicates the stent); F) Left anterior oblique angiographic view of the successful percutaneous intervention on the proximal RCA (arrow indicates the stent in the proximal RCA)

LCx, Left circumflex artery; LMCA, Left main coronary artery; PA, Pulmonary artery; RCA, Right coronary artery; RV, Right ventricle

A 68-year-old male patient was admitted to the emergency department with a complaint of anginal chest pains at rest of 2 hours’ duration. Electrocardiography showed ST-segment depressions in the inferior leads. The cardiac troponin I level was high (3.0 ng/mL, normal range between 0 and 0.1 ng/mL). There were no additional coronary artery risk factors, except for hypertension. Transthoracic echocardiography revealed mild inferior wall hypokinesia. With an initial diagnosis of non–ST-elevation myocardial infarction, coronary angiography was performed, which interestingly revealed that the left main coronary artery originated directly from the right sinus of Valsalva. In addition, there were critical stenoses in the left circumflex coronary artery and the right coronary artery (Figures 1A & 1B; Video 1). Cardiac computed tomography showed that there was no external compression or interarterial course for the anomalous coronary arteries (Figures 1C & 1D). With the aid of a Judkins right 6-F guiding catheter, a 2.5×15 mm XIENCE PRO Stent (Abbott Vascular, USA) was implanted to relieve a 90% occluded anomalous left circumflex coronary artery and a 3.0×16 mm PROMUS Element Stent (Boston Scientific, USA) was used to treat a 90% stenosis in the proximal right coronary artery (Figures 1E & 1F; Video 2). Fractional flow reserve was performed for suspicious critical ostial left anterior descending artery stenosis, and the result was noncritical. The patient was discharged without any complication and was followed up asymptomatically for 6 months.

***Case Report # 2***

**Figure 2:**
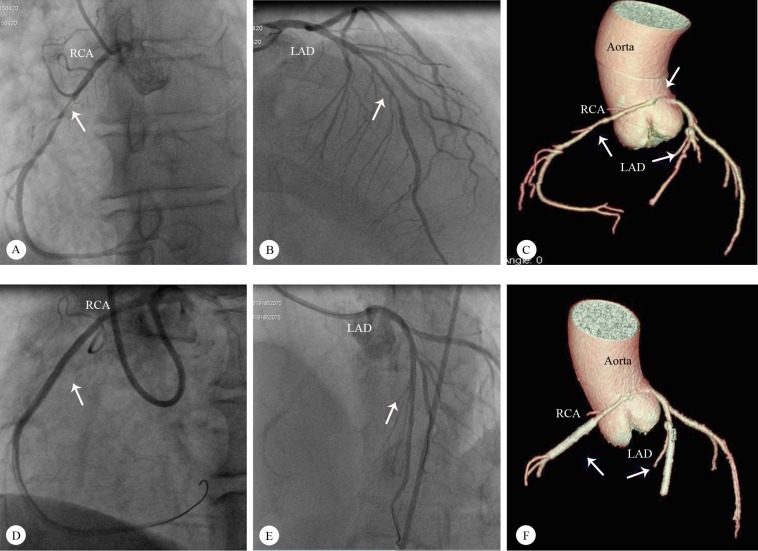
A) Left anterior oblique angiographic view of the RCA originating from the left sinus of Valsalva and a severe midportion RCA stenosis (arrow indicates the stenosis); B) Right anterior oblique cranial angiographic view of a severe midportion LAD stenosis (arrow indicates the stenosis); C) Computed tomographic left anterior angiographic imaging of the course of the RCA originating from the left sinus of Valsalva (arrow indicates the RCA origin and stenoses in the RCA and the LAD); D) Left anterior oblique angiographic view of the successful percutaneous intervention on the midportion of the RCA (arrow indicates the stent); E) Left anterior oblique cranial angiographic view of the successful percutaneous intervention on the LAD (arrow indicates the stent); F) Computed tomographic left anterior angiographic imaging of the stents in the LAD and the RCA 1 month after percutaneous intervention (arrow indicates the stent)

LAD, Left anterior descending artery; RCA, Right coronary artery

A 54-year-old male patient presented to the emergency department with a complaint of anginal chest pains during exercise increasing gradually over a period of 1 month. Electrocardiography showed an early repolarization pattern. Cardiac enzymes were normal. There was no risk factor for coronary artery disease. The exercise stress test was stopped early due to chest pains and significant ST-segment depressions in multiple leads. With an initial diagnosis of unstable angina pectoris, coronary angiography was performed and, interestingly, the right coronary artery was observed to originate directly from the left sinus of Valsalva. In addition, there were critical stenoses in the right coronary artery and the left anterior descending coronary artery (Figures 2A & 2B; Video 3). Cardiac computed tomography showed that the anomalous right coronary artery was passing between the aorta and the pulmonary artery with a slight decrease in vessel calibration during the interarterial course (Figure 2C). With the aid of an Amplatz left 6-F guiding catheter, a 2.75×33 mm Resolute Integrity Stent (Medtronic, USA) was implanted to relieve a 90% occluded anomalous right coronary artery. The left anterior descending coronary artery was cannulated with a Judkins left 6-F guiding catheter, and another 2.75×33 mm Resolute Integrity Stent (Medtronic, USA) was used to treat a 90% stenosis (Figures 2D & 2E; Video 4). The patient was discharged without any complication. Control cardiac computed tomography, performed 1 month later, showed that there was no compression by the aorta or the pulmonary artery on the stent implanted to the anomalous right coronary artery (Figure 2F). In addition, there was no evidence of ischemia at stress myocardial perfusion scintigraphy. The patient was followed up asymptomatically for 6 months.

## Discussion

The frequency of CAAs ranges from 0.21% to 5.79% according to angiography, computed tomography, magnetic resonance imaging, and autopsy data banks.^4^ There are origination, course, termination, and intrinsic structure anomalies and these anomalies may be associated with other congenital cardiovascular diseases such as the transposition of great vessels, bicuspid aortic valves, the tetralogy of Fallot, and arteriovenous fistulae.^1^^, ^^4^^, ^^5^ There is no consensus as to the clinical significance of CAAs. The most clinically important anomaly is the origin of the coronary artery from the opposite aortic root together with a passage between the aorta and the pulmonary artery (ie, interarterial course), causing myocardial ischemia, syncope, or sudden death during exercise.^1^^, ^^5^^-^^7^ Anomalous left and right coronary arteries originating from the contralateral sinus of Valsalva and containing an intramural segment coursing between the aorta and the main pulmonary artery can be seen occasionally as well.^5^^, ^^6^ Aberrant origins and courses of coronary arteries could make them more prone to atherosclerosis.^1^^, ^^3^

Although CAAs are frequently asymptomatic, they may cause chest pains, dyspnea, palpitation, syncope, sudden death, ventricular fibrillation, or myocardial infarction.^1^^, ^^8^^, ^^9^ Among the mechanisms explaining myocardial ischemia and sudden death due to CAAs are long courses of coronary arteries, angulation, intramural courses, cleft-like coronary artery orifices with dynamic obstructions, vasospasm, endothelial injury, and the compression of the aberrant artery between pulmonary and aortic roots, especially during exercise.^1^^, ^^6^^, ^^7^ Taylor et al^10^ showed that 25% of CAAs presented with sudden death and most of them were asymptomatic. Eckart et al^11^ revealed high sudden death risks in CAAs. Since they are associated with clinically important outcomes, CCAs should be evaluated systematically with different imaging modalities. **T**he main diagnostic tool is selective coronary angiography.^1^ Noninvasive imaging methods such as coronary computed tomography angiography or cardiac magnetic resonance imaging provide supplementary information.^1^^, ^^6^^, ^^9^ In addition, intravascular ultrasound can be preferred to evaluate the mechanisms responsible for ischemia.^6^

When CCAs present with atherosclerotic stenosis, treatment options include medical management, percutaneous interventions, and surgical operations. Percutaneous interventions have some difficulties including inadequate guiding-catheter support and the need for an experienced operator.^1^^, ^^4^ In the literature, increasing percutaneous interventions are encouraging in different coronary anomalies in our daily practice.^12^^, ^^13^ The most important problem is diagnosis due to the origin of the different ostia and also the guiding-catheter support during percutaneous interventions.^12^^, ^^14^ The Amplatz left catheter is useful for engaging an aberrant right coronary artery that originates from the midline of the aortic root and the anterior or horizontal takeoff in the right sinus of Valsalva. The Voda catheter is useful for engaging an aberrant originating coronary artery for horizontal and downward locations in the aortic root.^14^ Moreover, forming the Judkins right catheter by straightening its tip or increasing its curve and using buddy wires are other supporting techniques.^13^^, ^^14^ Our cases are interesting in that successful percutaneous interventions were provided by using different guiding catheters. Additionally, CAAs were evaluated by different imaging methods before and after an intervention.

## Conclusion

Percutaneous treatment for atherosclerotic occlusions in anomalous coronary arteries has some difficulties. In order to overcome some of these difficulties, we should evaluate coronary artery anomalies systematically with different imaging modalities. 

## Notes:

***This paper should be cited as: ***Akcay M, Camlıdag I, Gulel O. Percutaneous Coronary Intervention for Coronary Arteries with Origin and Course Anomalies: Case Reports and Literature Review. J Teh Univ Heart Ctr 2019;14(3):141-145.

## Notes:

***To watch the following videos, please refer to the relevant URLs: ***

http://jthc.tums.ac.ir/index.php/jthc/article/view/954/863

Video 1. Angiographic imaging of the left main coronary artery originating from the right sinus of Valsalva and severe left circumflex artery and right coronary artery stenoses

http://jthc.tums.ac.ir/index.php/jthc/article/view/954/864

Video 2. Angiographic imaging of the percutaneous intervention on the left circumflex artery and right coronary artery stenoses

http://jthc.tums.ac.ir/index.php/jthc/article/view/954/865

Video 3. Angiographic imaging of the right coronary artery originating from the left sinus of Valsalva and severe Left anterior descending artery and right coronary artery stenoses

http://jthc.tums.ac.ir/index.php/jthc/article/view/954/866

Video 4. Angiographic imaging of the percutaneous intervention on the left anterior descending artery and right coronary artery stenoses
